# Perioperative adrenal insufficiency presenting as refractory hypotension: A CARE-compliant article

**DOI:** 10.1097/MD.0000000000050998

**Published:** 2026-10-02

**Authors:** Ye Sull Kim, Seung Park, Yejin Song, Aram Doo

**Affiliations:** a Department of Anesthesiology and Pain Medicine, Jeonbuk National University Medical School, Jeonju, Jeollabuk-Do, South Korea; b Research Institute of Clinical Medicine of Jeonbuk National University-Biomedical Research Institute of Jeonbuk National University Hospital, Jeonju, South Korea.

**Keywords:** adrenal insufficiency, corticosteroids, general anesthesia, hypotension, perioperative complications

## Abstract

**Rationale:**

Adrenal insufficiency (AI) is a rare but potentially life-threatening cause of perioperative hemodynamic instability characterized by an inadequate cortisol response to physiological stress.

**Patient concerns:**

We report a case of a 77-year-old woman undergoing oblique lumbar interbody fusion who developed profound intraoperative hypotension refractory to fluid resuscitation and multiple vasopressors. Notably, she had a history of corticosteroid therapy for rheumatoid arthritis that had been discontinued nearly 3 decades earlier. She also exhibited extreme skin fragility.

**Diagnoses:**

AI was clinically suspected based on persistent vasopressor-resistant hypotension, a remote history of corticosteroid therapy, and extreme skin fragility.

**Interventions:**

Suspecting AI, intravenous hydrocortisone was administered postoperatively, resulting in rapid hemodynamic stabilization and subsequent weaning of vasopressors.

**Outcomes:**

Pre- and postoperative biochemical testing was inconclusive for definitive AI. However, the clinical presentation and dramatic response to steroid administration strongly suggested surgical stress-related AI. The patient was discharged without any complications.

**Lessons:**

This case underscores the importance of maintaining a high index of suspicion for AI in patients with unexplained, vasopressor-resistant hypotension, even those with a remote history of corticosteroid use, as timely empirical treatment can be life-saving.

## 1. Introduction

Adrenal insufficiency (AI) is defined as inadequate production or secretion of glucocorticoids, primarily cortisol, by the adrenal cortex, leading to an impaired ability to maintain homeostasis during physiologic stress.^[1]^ Cortisol plays a crucial role in maintaining vascular tone, electrolyte balance, and cardiovascular stability.^[2–4]^ Therefore, an insufficient adrenal response during anesthesia and surgery can result in profound, refractory hypotension and cardiovascular collapse if not promptly recognized and treated.

AI is classified into primary, secondary, and tertiary types according to the level of dysfunction along the hypothalamic–pituitary–adrenal (HPA) axis.^[5]^ Primary AI results from intrinsic adrenal gland failure, while secondary AI is caused by pituitary pathology. Tertiary AI results from hypothalamic suppression due to chronic exogenous glucocorticoid therapy, leading to decreased corticotropin-releasing hormone secretion and subsequent reduction in adrenocorticotropic hormone (ACTH) release. It is the most common cause of AI in clinical practice, especially among patients with prolonged glucocorticoid therapy.^[6]^

During major surgical procedures, cortisol secretion in healthy individuals may increase up to 5- to 10-fold, equivalent to approximately 75 to 150 mg of hydrocortisone per day.^[7]^ This stress response is essential for maintaining hemodynamic stability and metabolic homeostasis. When the HPA axis is suppressed – particularly in patients with prior glucocorticoid exposure – the expected cortisol surge may fail to occur, thereby predisposing patients to adrenal crisis and resulting in refractory hypotension unresponsive to fluid resuscitation and vasopressors.^[8]^ Current recommendations suggest perioperative glucocorticoid supplementation for patients with known or suspected HPA-axis suppression, but not routinely for those with remote glucocorticoid exposure.

The present case highlights the importance of recognizing perioperative AI as a potential cause of vasopressor-resistant hypotension and emphasizes the need for timely steroid administration when clinical suspicion arises. Through this report, we review the mechanisms, diagnostic considerations, and anesthetic implications of perioperative AI.

## 2. Case presentation

Written informed consent for publication of this case was obtained from the patient. A 77-year-old woman (155 cm in height and 56 kg in weight) was scheduled to undergo oblique lumbar interbody fusion at L3–L5 due to severe lumbar spinal stenosis. Her medical history included hypertension and diabetes mellitus, for which she had been receiving ongoing medical treatment for approximately 10 years. She had also been diagnosed with rheumatoid arthritis during young adulthood and had a history of long-term glucocorticoid therapy for approximately 10 years, which had been discontinued 30 years prior to surgery.

Baseline laboratory analyses, including routine hematologic and biochemical tests, were within normal ranges. Chest radiography showed findings consistent with old, healed pulmonary tuberculosis in both lungs. Electrocardiography revealed sinus tachycardia with a rate of 104 beats per minute. On preoperative evaluation, the patient’s skin appeared extremely thin and fragile, particularly on the face and extremities, with easy bruising or purpura observed in several areas, suggestive of steroid-related cutaneous changes. However, dynamic testing with the corticotropin stimulation test and preoperative corticosteroid supplementation were not planned, as the preoperative serum cortisol level measured at 16:30 was 3.9 µg/dL, which was within the normal reference range,^[9]^ and the history of chronic glucocorticoid use was considered sufficiently remote to allow restorage of HPA-axis function.

On the day of surgery, antihypertensive medication containing an angiotensin receptor blocker was withheld to minimize the risk of refractory intraoperative hypotension. Anesthesia was induced using target-controlled infusion of propofol and remifentanil, and rocuronium 40 mg was administered to facilitate endotracheal intubation. The patient was ventilated with an oxygen–air mixture (fraction of inspired oxygen = 0.5). During the anesthesia induction, vital signs remained stable.

The patient’s vital signs remained stable during the early intraoperative period; however, a gradual decline in blood pressure was observed approximately 1.5 hours after the initiation of the surgery. Intermittent intravenous administration of ephedrine produced only a minimal hemodynamic response. Despite reducing the effect-site concentrations of propofol and remifentanil to 1.5 μg/mL and 0.5 ng/mL, respectively (Table 1), and initiating vasoactive support with dopamine (5–15 μg/kg/min) and norepinephrine (up to 7.5 μg/min), the patient’s systolic blood pressure persisted at approximately 70 mm Hg, consistent with vasopressor-resistant hypotension (Fig. 1). Hypotension persisted despite discontinuation of the anesthetic agent at the conclusion of surgery. Potential causes of refractory hypotension were considered. Intraoperative blood loss was minimal, with no evidence of significant hemorrhage contributing to the hypotension, and the total volume of administered intravenous fluid was 2000 mL. Anaphylaxis was unlikely in the absence of cutaneous manifestations, laryngeal edema, or bronchospasm. Furthermore, there was no clinical or laboratory evidence of sepsis or other apparent causes of vasoplegia.

**Table 1:** Anesthetic consumption, bispectral index, and analgesia nociception index.

	AI	SI	SI-30	SI-60	SI-90	SI-120	SC
Propofol (µg/mL)	4.0	3.0	2.5	2.0	2.0	1.5	1.5
Remifentanil (ng/mL)	3.0	1.5	0.5	1.5	0.5	0.5	0.5
BIS	90	41	40	48	50	68	65
ANI	85	55	48	67	65	63	52

AI = anesthesia induction, ANI = analgesia nociception index, BIS = bispectral index, SC = skin closure, SI = skin incision, SI-30 = 30 minutes after skin incision, SI-60 = 60 minutes after skin incision, SI-90 = 90 minutes after skin incision, SI-120 = 120 minutes after skin incision.

**Figure 1.**
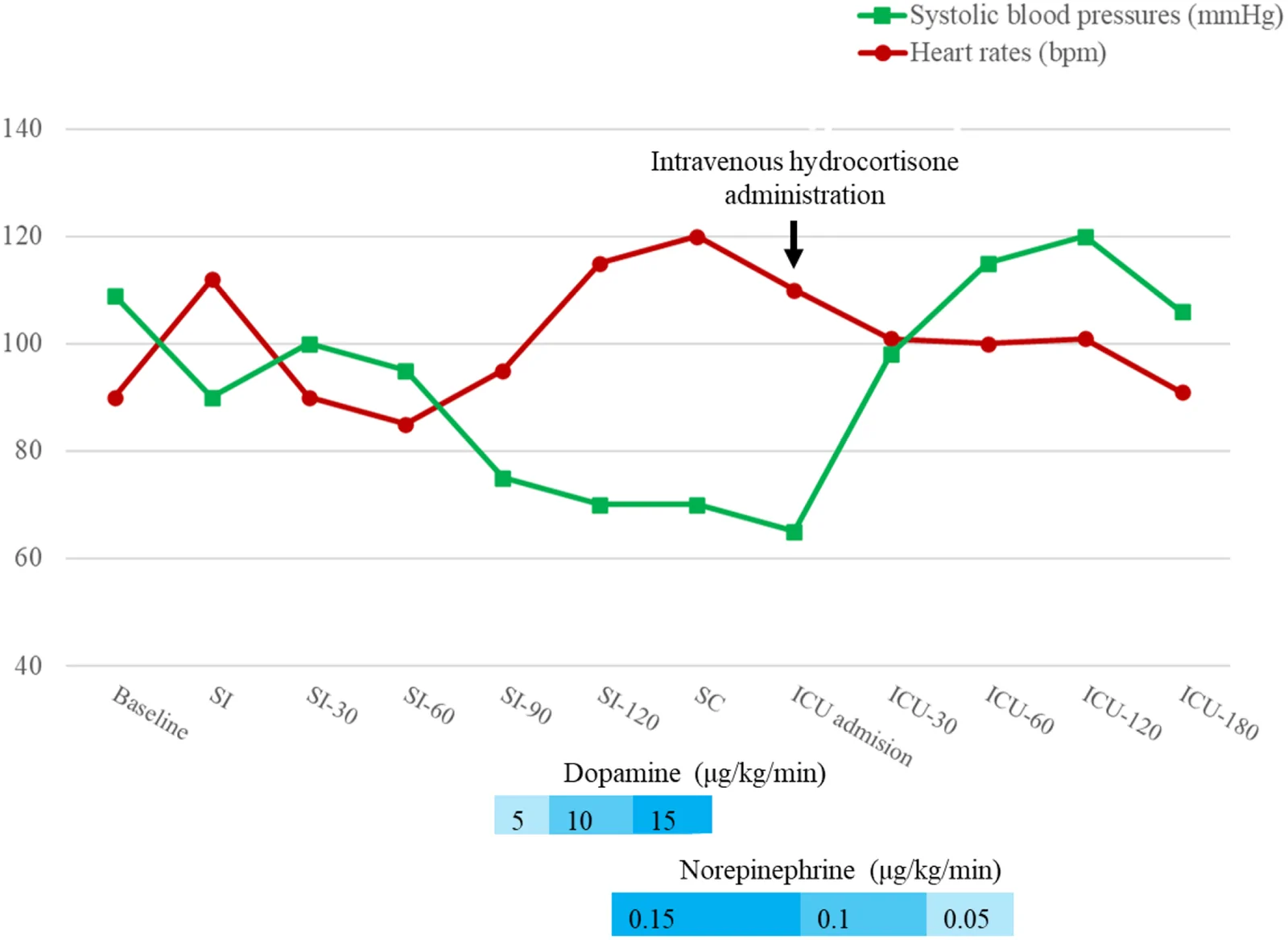
Changes in systolic blood pressure and heart rate. ICU = intensive care unit, ICU-30 = 30 minutes after ICU admission, ICU-60 = 60 minutes after ICU admission, ICU-120 = 120 minutes after ICU admission, ICU-180 = 180 minutes after ICU admission, SI = skin incision, SI-30 = 30 minutes after SI, SI-60 = 60 minutes after SI, SI-90 = 90 minutes after SI, SI-120 = 120 minutes after SI, SC = skin closure.

Given the refractory hypotension and the patient’s clinical history, intraoperative AI was suspected, and the patient was transferred to the intensive care unit (ICU) for further management. During the event, arterial blood gas analysis and laboratory testing showed a pH of 7.346, base excess of −3.0 mmol/L, lactate of 1.0 mmol/L, sodium of 140 mmol/L, potassium of 2.8 mmol/L, and glucose of 208 mg/dL. Following intravenous administration of hydrocortisone 100 mg in the ICU, the patient’s blood pressure rapidly stabilized within 30 minutes, allowing gradual tapering and subsequent discontinuation of vasopressor support (Fig. 1).

Transthoracic echocardiography, which was performed for the differential diagnosis of profound hypotension, revealed normal left ventricular systolic function with an ejection fraction of 62%, diastolic dysfunction grade 1, and mild degenerative aortic stenosis. No definitive cardiac etiology was identified on cardiology consultation. A retrospective review of her medical records after surgery revealed that she had recently undergone several epidural blocks for lumbar spinal stenosis; however, whether glucocorticoids had been administered during these procedures could not be confirmed. The patient was transferred to the general ward in stable condition without any significant postoperative complications on the following day.

Meanwhile, marked skin fragility with multiple purpura and bullae was noted on both forearms before surgery. Accordingly, all monitoring devices and fixation materials were applied with soft, nonadhesive tape to minimize skin injury. Nevertheless, during patient repositioning for the oblique lumbar interbody fusion procedure, skin erosion developed at both forearms, with exposure of the subcutaneous tissue despite careful handling (Fig. 2).

**Figure 2.**
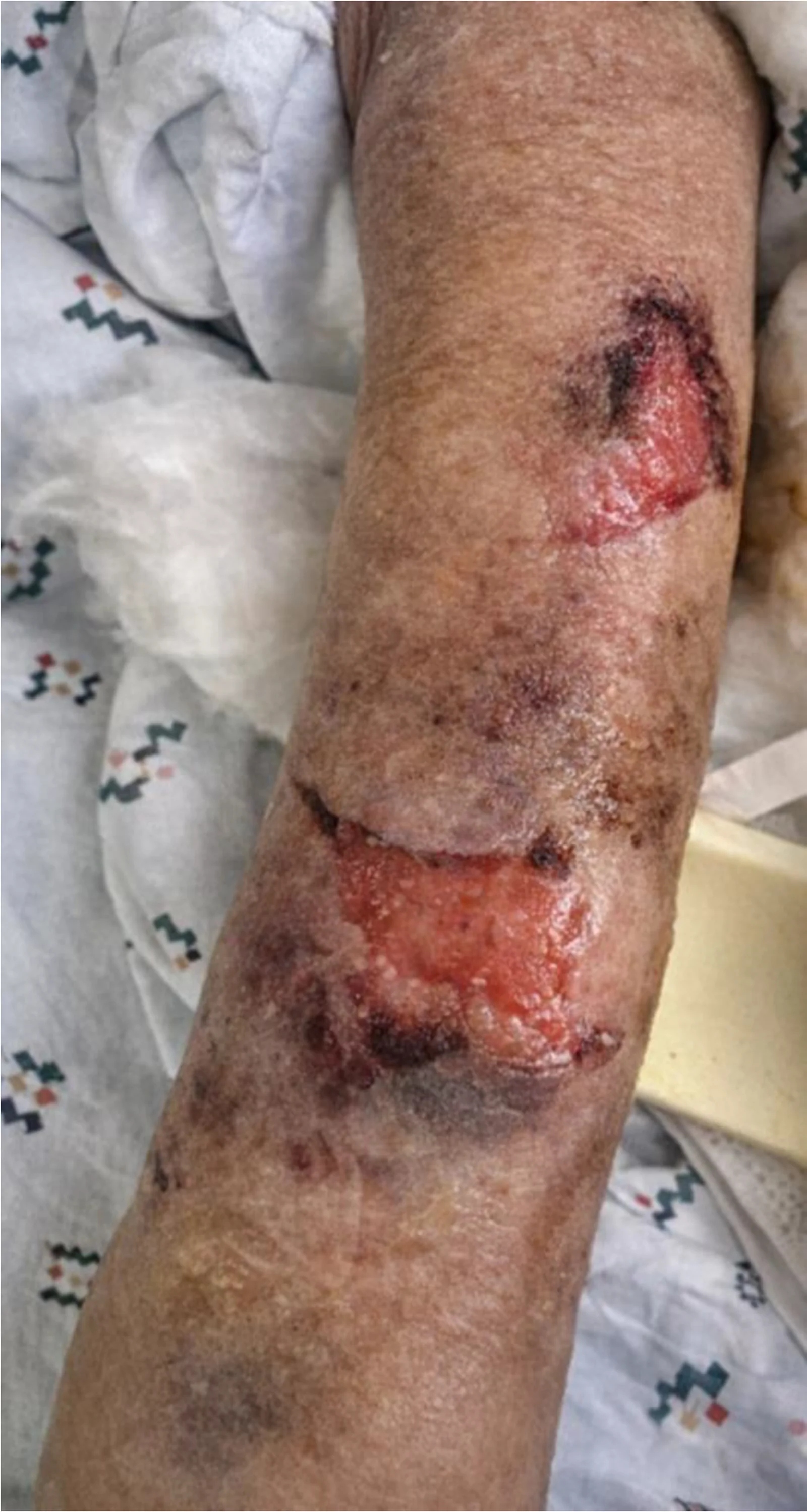
Marked skin fragility with forearm abrasion.

## 3. Discussion

This case report underscores the importance of considering perioperative AI as a potential cause of unexplained, vasopressor-resistant hypotension during major surgery. Although recovery of the HPA axis generally occurs after glucocorticoid withdrawal, the time course of recovery varies among individuals. Accordingly, prompt recognition and timely administration of corticosteroids are crucial when AI is clinically suspected. Because the patient in this case had discontinued steroid treatment several decades earlier, the possibility of AI was initially overlooked.

Several factors in this patient raised clinical suspicion for perioperative AI. First, she had a remote history of long-term glucocorticoid therapy for rheumatoid arthritis. Although persistent HPA-axis suppression attributable to glucocorticoid therapy discontinued 3 decades earlier cannot be established, this history was considered in the overall clinical assessment. Second, a retrospective review of her medical history after surgery revealed that she had recently undergone several epidural blocks for the treatment of lumbar spinal stenosis. Although it was unclear whether glucocorticoids had been administered during these procedures, repeated exposure to epidural steroids cannot be excluded. If glucocorticoids had been administered, such exposure could have represented an additional potential contributor to HPA-axis suppression.

In addition, the presence of markedly thin and fragile skin with easy bruising might suggest chronic glucocorticoid-related systemic effects rather than age-related changes alone. Preoperatively, her serum cortisol level measured in the late afternoon was at the lower limit of the reference range. However, interpretation of this value was limited by the timing of sampling because cortisol secretion follows a circadian rhythm and normally declines throughout the day. Therefore, a single late-afternoon cortisol measurement cannot reliably assess HPA-axis function or exclude AI. Dynamic endocrine testing, such as an ACTH stimulation test (cosyntropin stimulation test), might have been useful for the preoperative assessment of adrenal function in this patient.^[10,11]^ Finally, the prompt resolution of vasopressor-resistant hypotension following intravenous hydrocortisone administration strongly supports the likelihood of AI in this case.

In clinical practice, physicians generally prevent the development of adrenal crisis by administering intravenous hydrocortisone before major surgery in patients who have received long-term or high-dose glucocorticoid therapy. In contrast, a remote history of glucocorticoid therapy is often overlooked. However, this case warns that the recovery of the HPA axis cannot be assumed solely based on the duration of cessation. Prolonged glucocorticoid use can induce persistent adrenal cortical atrophy through suppression of ACTH-dependent trophic stimulation and downregulation of key steroidogenic enzymes, as demonstrated in experimental models.^[12,13]^ Importantly, the recovery of adrenal morphology and steroidogenic capacity after steroid withdrawal is frequently delayed and exhibits substantial interindividual variability, even after hypothalamic–pituitary signaling has normalized.^[13,14]^ Notably, recovery of adrenal structure and function after steroid withdrawal is frequently delayed and highly variable among individuals, and HPA-axis recovery may remain incomplete for years,^[15,16]^ with clinically relevant AI becoming apparent only during periods of significant physiologic stress. Accordingly, clinicians should maintain a high index of suspicion for adrenal suppression in patients with unexplained intraoperative hypotension.

The diagnosis of AI relies on both basal hormone levels and dynamic testing. A morning (08:00) serum cortisol level below 3 µg/dL is highly suggestive of AI, while a level above 15 to 18 µg/dL generally excludes it.^[17]^ Values in the intermediate range (3–15 µg/dL) require further evaluation with an ACTH (cosyntropin) stimulation test. In secondary or tertiary forms, ACTH levels are typically low or inappropriately normal despite low cortisol. However, during acute illness or surgery, interpretation of cortisol and ACTH values can be challenging, and clinical context should guide management. Meanwhile, hyponatremia and hyperkalemia might be key laboratory findings in primary AI.^[3]^ However, normal serum sodium does not exclude AI, especially in secondary or tertiary types of AI, because mineralocorticoid activity would be preserved. In this patient, the serum sodium was within the normal range both pre- and postoperatively.

Anesthetic implications include preoperative identification of patients at risk – those with current or prior glucocorticoid use. Patients receiving glucocorticoid therapy equivalent to prednisolone ≥5 mg/d are considered at risk of developing AI perioperatively.^[11]^ In patients with possible AI, hydrocortisone 100 mg should be given intravenously at the induction of anesthesia, followed by 50 mg every 6 to 8 hours or continuous infusion (200 mg/d) during major surgical stress.^[10,11]^ If severe AI or adrenal crisis is suspected intraoperatively in the absence of prophylactic steroid administration, hydrocortisone should be administered promptly without waiting for diagnostic confirmation.^[10]^ Once hemodynamic stability is achieved, glucocorticoids should be gradually tapered to physiologic replacement doses.

This case has several limitations. ACTH levels were not measured, and dynamic endocrine testing was not performed; therefore, AI could not be biochemically confirmed or further classified. In addition, although the clinical presentation and rapid hemodynamic response to hydrocortisone supported AI as the most likely diagnosis, a definitive causal relationship between adrenal dysfunction and the refractory hypotension could not be established.

## 4. Conclusion

In conclusion, AI was considered the most likely diagnosis for the unexplained, vasopressor-resistant perioperative hypotension in this case, although it could not be definitively confirmed. In such settings, biochemical testing is often impractical or delayed, and clinical features – including hemodynamic instability refractory to conventional therapy and prompt responsiveness to corticosteroids – may be critical in guiding management. Given the potentially life-threatening consequences of delayed treatment, early empirical administration of hydrocortisone should be considered when AI is clinically suspected.

## Author contributions

**Conceptualization:** Aram Doo.

**Data curation:** Seung Park.

**Supervision:** Aram Doo.

**Writing – original draft:** Ye Sull Kim.

**Writing – review & editing:** Ye Sull Kim, Seung Park, Yejin Song, and Aram Doo.
